# Do the majority of Malaysian women have dense breasts on mammogram?†

**DOI:** 10.2349/biij.7.2.e14

**Published:** 2011-04-01

**Authors:** MA Zulfiqar, I Rohazly, MA Rahmah

**Affiliations:** 1 Department of Radiology, Faculty of Medicine, Universiti Kebangsaan Malaysia Medical Centre, Kuala Lumpur, Malaysia; 2 Department of Community Health, Faculty of Medicine, Universiti Kebangsaan Malaysia Medical Centre, Kuala Lumpur, Malaysia

**Keywords:** Mammography, breast parenchymal patterns, breast density

## Abstract

**Purpose:**

To determine: (i) the mammographic parenchymal patterns in Malaysian women and whether the breasts are dense on mammogram; (ii) the effect of age on breast density; (iii) the effect of parity on breast density; (iv) the difference in breast parenchymal patterns among the major races of women in Malaysia.

**Methods:**

This was a descriptive cross-sectional study of 1,784 patients (981 Malays, 571 Chinese, 214 Indians and 18 others) who had undergone mammography during the 1-year study period. Majority of women (41.7%) were aged between 51 and 60 years and majority (43%) had 3–4 children. The Tabar classification (Pattern I - V) was used to evaluate breast parenchymal patterns on mammogram. Tabar Pattern I was further divided into 3 sub-groups (Pattern IA, IB, and IC). The different patterns were then grouped into dense (IB, IC, IV, V) and not dense (IA, II, III) breasts. The SPSS package was used for statistical analysis.

**Results:**

Majority (59%) of Malaysian women had dense breasts (Pattern IB 29%, IC 20%, IV 5%, and V 5%) and 41% did not have dense breasts (Pattern IA 28%, II 6%, and III 7%). Age and parity were inversely related to breast density (p < 0.0001). Chinese women (65.7%) had the highest percentage of dense breasts (p = 0.69, odds ratio = 1.22), followed by the Indians (57.2%) and the Malays (50.5%).

**Conclusion:**

Majority of women had dense breasts but Pattern IV, which has been associated with increased risk of breast cancer, was seen in only 5% of the women. The breast density reduced steadily with increasing age and parity. There was no statistically significant difference in breast density in the three main races.

## INTRODUCTION

### Mammographic parenchymal patterns and density

The terms mammographic parenchymal patterns and mammographic density are widely used to describe the proportion of dense to lucent areas in the breast as seen on mammogram. The appearance of breast tissue on mammogram varies according to its composition [1]. The glandular tissue that comprises of the terminal duct lobular units (TDLU) is dense and nodular on mammogram. Ducts, vessels and ligaments are dense and curvilinear. The fibrous tissue is dense and homogenous. Fat is lucent.

### Rationale of the study

Mammographic density has been reported as a risk factor for breast carcinoma [2]. The anecdotal findings of radiologists involved in breast imaging are that the majority of Malaysians have dense breasts on mammogram regardless of age and parity. If this observation is true, then there would be a high incidence of breast carcinoma in Malaysia. The breast carcinoma age-standardised incidence (ASR) in Malaysia is 47.4 per 100,000 [3]. This is significantly lower compared to the breast carcinoma incidence in United Kingdom (ASR of 74.4 per 100,000) [3] and United States (ASR of 92.1 per 100,000) [3]. It is therefore interesting to determine whether the observation that Malaysian women have dense breasts is accurate. The purpose of this study was to determine: (i) the mammographic parenchymal patterns in Malaysian women, and whether the breasts are dense on mammogram; (ii) the effects of age; (iii) the effects of parity; and (iv) the difference in parenchymal patterns among the major races.

## METHODS

This study was approved by the hospital technical and ethical committee. Patient informed consent was not obtained as this was a retrospective review.

### Subjects

This was a descriptive cross-sectional study of 1,784 women who had mammography at our hospital over a one-year period from September 2004 to August 2005. Those who had breast cancer and those on hormone replacement therapy were also included. Women whose mammograms were not available for review were excluded.

### Image acquisition

Two-view (cranial-caudal and medial-lateral oblique) film-screen mammography examinations were performed using the Siemens Mammomat 3000 (Germany).

### Assessment of mammographic parenchymal pattern and density

**Figure 1:**
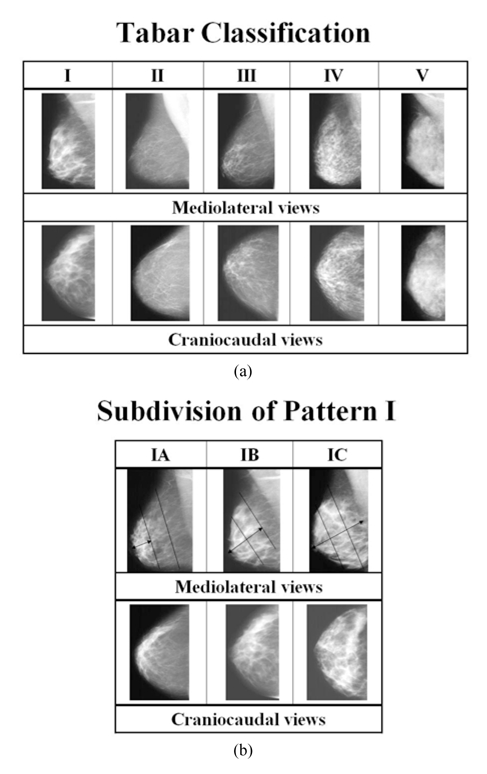
a) Tabar Classification of types of parenchymal patterns as seen on mammogram; b) subdivision of Tabar Pattern I into 3 levels of density.

The method of classification based on anatomic-mammographic correlation developed by Tabar (Figure 1a) was used [1]. Pattern I shows (i) scalloped contour and Cooper ligaments; (ii) evenly scattered terminal duct lobular units (TDLU) that appear as 1-2 mm nodular densities; and (iii) oval-shaped lucent intra-mammary fat lobules. Pattern II shows (i) mainly fat; and (ii) linear opacities due to ligaments, ducts, or vessels. Pattern III shows (i) mainly fat; and (ii) retro-areolar ducts. Pattern IV shows (i) convex contour; and (ii) TDLU larger than 1-2 mm due to proliferating glandular structures. Pattern V shows (i) smooth convex contour; and (ii) homogenous opacity due to fibrous tissue.

Tabar Pattern I was found to have a wide spectrum of density and therefore it was further divided into 3 sub-groups (Figure 1b). In Pattern IA, glandular tissue (TDLU) occupies the distal one-third of the breast. In Pattern IB, glandular tissue occupies the distal two-thirds of the breast. In Pattern IC, glandular tissue occupies the entire breast.

Dense breast on mammogram was defined as at least 50% of the breast containing dense tissue. Therefore, Pattern II, III, and IA were classified as not dense. Pattern IB, IC, IV, and V were classified as dense.

The cranial-caudal and medial-lateral oblique views were viewed by a radiologist who was blinded to the women’s clinical data.

### Statistical analysis

Prevalence odds ratios (OR) were used to express the degree of association between mammographic patterns and the three selected factors, i.e. age, parity and race. A binary logistic regression model was used to allow for the effects of several potential confounders. Results were considered as statistically significant if the p-value was 0.05 or less and 95% confidence intervals (CI) were reported throughout the paper. The multiple logistic regression analyses were performed using the SPSS package.

## RESULTS

There were 95 (5%) women below the age of 40, 526 (30%) who were 41–50 years old, 840 (47%) who were 51–60 years old and 323 (18%) women older than 60 years. There were 238 (13%) nulliparous women, 481 (27%) had 1 or 2 children, 775 (44%) had 3 or 4 children, and 290 (16%) had more than 4 children. There were 981 (55%) Malay women, 571 (32%) Chinese, 214 (12%) Indians and 18 (1%) were women of other races.

**Figure 2:**
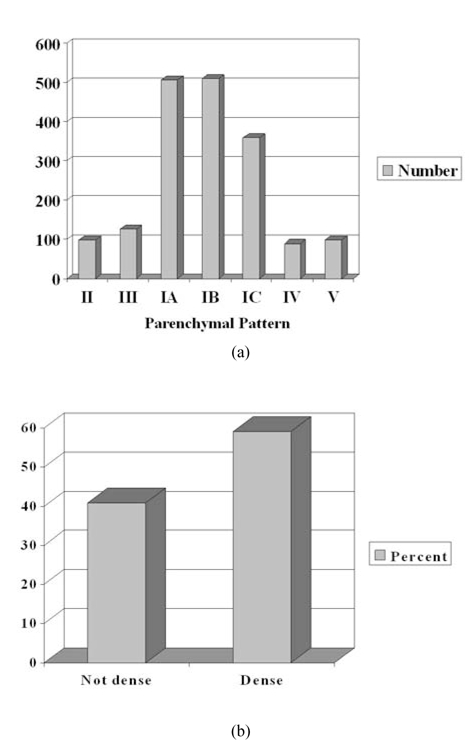
a) Distribution of number of women with the various types of parenchymal pattern. The order of parenchymal pattern from left to right is from least dense to most dense; b) percentage of women with non-dense and dense mammograms.

### Parenchymal pattern and breast density

Majority (77%) had Pattern I (Figure 2a). Patterns II and III were seen in about 6% and 7% of women, respectively. Patterns IV and V contributed about 5% each. For Pattern I, 28% were not dense (Pattern IA) and 49% were dense (Pattern IB and IC). There were 729 women with non-dense breasts and 1,055 women with dense breasts (Figure 2b).

### Age and breast density

**Figure 3:**
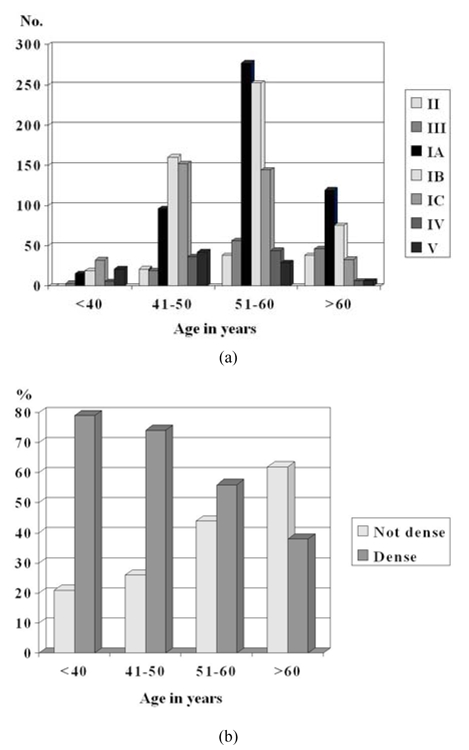
a) Distribution of number of women with the various types of parenchymal pattern according to age. (The bars are arranged from left to right in order of increasing breast density i.e. Pattern II, III, IA, IB, IC, IV, and V); b) percentage of women with non-dense and dense mammograms according to age.

Pattern I sub-type that predominated in each age cohort reduced in density (from Pattern IC to IA) as the age increased (Figure 3a). In the cohort of those aged 40 and below, Pattern IC was predominant (44%). In the cohort of those 41–50 years old, Pattern IB was predominant (40%). The majority of those in the cohorts of 51–60 years and above 60 years had Pattern IA (41% and 54% respectively).

Pattern II that represented complete fatty replacement was seen predominantly in the two cohorts of 51–60 years and above 60 years (39% and 35% respectively). The highest occurrence of Pattern IV was in the cohort of 51–60 years (49%) while the highest occurrence of Pattern V was seen in the cohort of 41–50 years (42%). Breast density reduced with increasing age (Figure 3b). The cohort of 40 years and below had the highest odds of having dense breasts, OR 2.72 (p < 0.001).

### Parity and breast density

**Figure 4:**
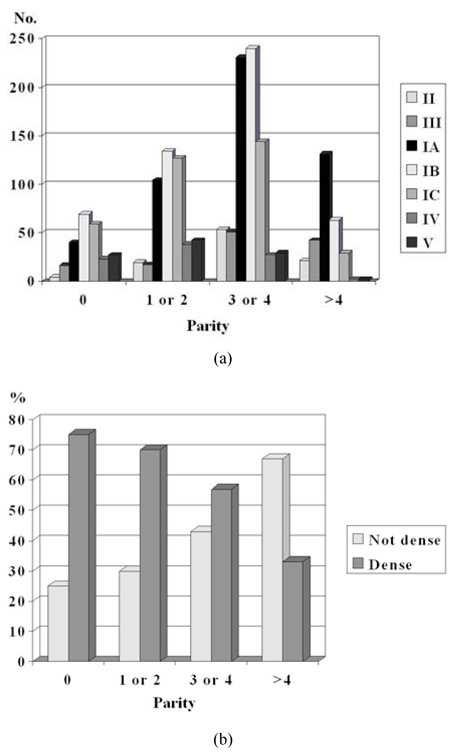
a) Distribution of number of women with the various types of parenchymal patterns according to parity. (The bars are arranged from left to right in order of increasing breast density i.e. Pattern II, III, IA, IB, IC, IV, and V); b) percentage of women with non-dense and dense mammograms according to parity.

The majority of women who were nulliparous and those with 1 or 2 children had Pattern IB (28%) and IC (26%) (Figure 4a). The majority of those with 3 or 4 children had Pattern IA and IB (30% and 31%, respectively). The majority of those with 5 or more children had Pattern IA (46%). The majority of women with dense Pattern IV and V had 1 or 2 children (42% each). Only a small percentage of those with more than 4 children had these patterns (2% each). In the different age groups, the percentage of those with dense breasts reduced as the number of children increased (Figure 4b).

There was an inverse association between parity and breast density. Nulliparous women had the highest odds of having dense breasts, OR 2.18 (p < 0.0001).

### Ethnicity and density

The majority with Pattern II breasts that had undergone complete fatty replacement were Malay women (61%). The occurrence of Pattern II was similar amongst the Chinese and Indians (19% and 18%, respectively).

**Figure 5:**
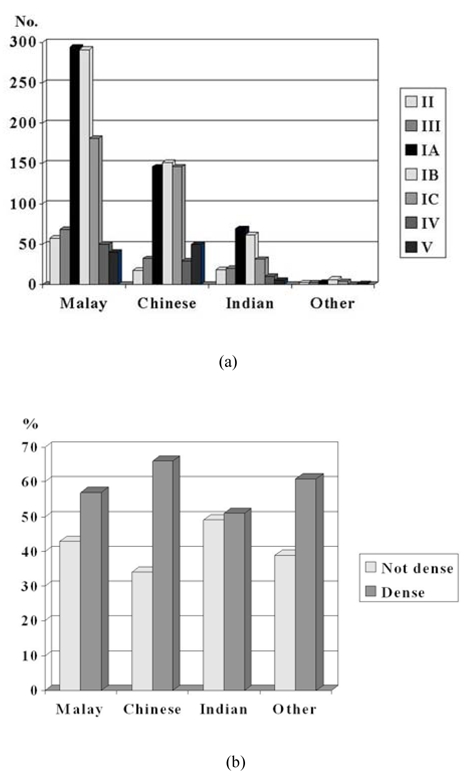
a) Distribution of number of women with the various types of parenchymal patterns according to ethnicity. (The bars are arranged from left to right in order of increasing breast density i.e. Pattern II, III, IA, IB, IC, IV, and V); b) percentage of women with non-dense and dense mammograms according to race.

In Malays and Indians, non-dense Pattern IA was predominant (30% each) (Figure 5a). The majority of Chinese had dense Pattern IB (26%) and IC (25%). Only a small percentage of Indian women had dense Pattern IV and V. Pattern IV was dominated by Malay women (56%), followed by Chinese (33%) and Indians (5%). For Pattern V, Chinese women dominated (51%), followed by Malays (43%) and Indians (5%).

Ethnic Chinese had the highest percentage of dense breasts (66%), followed by Malays (57%), and then Indians (51%) (Figure 5b). The other races were too few in number and too diverse to take into consideration. Chinese women had the highest odds of having dense breasts, OR 1.22. However, this finding was not statistically significant, p = 0.69.

## DISCUSSION

### Parenchymal pattern and breast density

Mammographic density has been reported as a risk factor for breast carcinoma [2, 4–8]. A systematic review and meta-analysis of well-conducted studies concluded that breast density is one of the strongest risk factors for breast cancer [2]. The review also refutes the suggestion that the association is an artefact of masking bias or that it is only present in a restricted age range.

It has been found that the prevalence of breast cancer is more than twice as high in women with Pattern IV compared with women with Pattern I-III [9]. The association between increased risks of breast cancer with Pattern IV has also been reported in an Asian population [10].

Our study showed that the majority of women had dense breasts but this was due to Pattern IB and IC, not due to Pattern IV that has been associated with increased risk of breast cancer [9–11]. The majority of our study population had Pattern I. Comparison with studies conducted in Singapore (Asian population) [10] and in Norway (Western population) [11] showed similar findings that the majority had Pattern I. The incidence of Pattern IV in Singapore and Norway is double that in Malaysia. However, this higher incidence of Pattern IV does not reflect the breast cancer incidence, as the incidence in Singapore [3] is almost similar to that of Malaysia [3], while the incidence in Norway [12] is almost double. This highlights the fact that there are multiple risk factors involved in breast cancer occurrence and it should not be correlated with breast density alone.

The incidence of dense breasts in our population is important to note, even though it is due to Pattern IB and IC. This is because dense breasts raise the possibility of missing early lesions that are small. Therefore, radiologists involved with breast imaging should remember to use ultrasound as an adjunct to mammography of dense breasts.

### Age and breast density

In post-menopausal breast involution, the lobules reduce in size, cells lining the acinar diminish, ductal epithelium atrophies and there is regression of adjacent connective tissue. These fibroglandular tissues are gradually replaced by fat [13]. Involutional structural changes may begin as early as 30 years old when the follicular-stimulating hormone (FSH) levels begin to rise progressively till menopause.

Screening mammograms taken at regular intervals have documented the transition from predominantly fibroglandular tissue (Pattern I) to predominantly fat tissue (Pattern II and III) [1].

Our study showed a similar decline in radiographic density with increasing age as reported in previous studies. This decline in density was best seen in Pattern I where the transition of pattern predominance from IC to IA was seen with progressively increasing age.

The majority of women with Pattern IV in our study were in the cohort of 51–60 years. It is interesting to note that it is this age group that leads the breast cancer incidence in our country [3].

It has been noted that Pattern IV appears resistant to the process of involution [1, 9]. The numerous enlarged nodular opacities in Pattern IV are due to proliferating glandular tissue (TDLU). Breast cancer is said to arise in the TDLU [13]. It is logical to assume that the abundance of TDLU that do not involute with age would increase the risk of developing cancer.

### Parity and breast density

During pregnancy the lobules increase in size and there is proliferation of lobular acini [13]. When lactation ceases, the breast undergoes a degree of involution. Successive pregnancies and lactation result in further involution and replacement of fibroglandular tissues with fat.

Our study showed a similar decline in radiographic density with increasing parity as previously reported. This decline in density was best seen in Pattern I where there was a predominance of Pattern IB and IC in the nulliparous and low parity groups to predominance of Pattern IA and IB in those with 3 and more children.

### Ethnicity and breast density

The finding that the ethnic Chinese had the highest odds of having dense breasts was not statistically significant. However, it is interesting to note that breast cancer incidence is highest amongst the ethnic Chinese in Malaysia [3]. The ASR according to ethnic groups was reported as: Chinese 59.9/100,000; Indians 54.2/100,000; and Malays 34.9/100,000 [3].

Ethnic Indians, who had the lowest percentage of dense breasts, came second after the Chinese in the incidence of breast cancer. Pattern IV was highest amongst Malay women who had the lowest incidence of breast cancer. These findings indicate that in our study population, breast parenchymal pattern and density were not good indicators of breast cancer risk.

### Limitations of the study

This study used a combination of qualitative and quantitative assessment. The mammograms were initially qualitatively classified according to Tabar patterns. Pattern IB, IC, IV, and V were then classified as dense because at least 50% of the breast contained dense tissue. This method of subjective assessment has several limitations, the most important being the low reproducibility. Two readers would have reduced subjectivity. However, at the time the study was conducted, there was only one trained reader available. There have been reports that quantitative assessment is superior to qualitative. However, a report comparing two quantitative (Cumulus and Madena software) and two qualitative (Wolfe and Tabar) methods showed the same overall associations between breast density and the risk factors for breast cancer [14].

The hospital at which the study was conducted is not a breast screening centre and women on hormone replacement therapy (HRT) had been included in this study. The use of HRT may influence breast density. However, an earlier study at the same centre showed that HRT did not cause any significant increase in breast density [15]. The inclusion of both pre- and post-menopausal women is another potential confounder.

Other potential factors that could affect mammographic patterns were not documented in this study. For example, age at menarche, age at first childbirth, body mass index (BMI) and height could all play a role in determining parenchymal patterns and breast density.

Finally, the women involved in this study were not truly representative of Malaysian women as the study population was based in an urban setting. Dietary habits, different lifestyles and levels of education could all contribute to differences in mammographic densities.

## CONCLUSION

The majority of women in this study population (59%) had dense breasts (Pattern IB, IC, IV, and V). Pattern IV, which has been associated with increased risk of breast cancer, was seen in only 5% of the women. Breast density reduced steadily with increasing age and parity. There was no statistically significant difference in breast density among the 3 major races

## References

[1] Tabar L, Tot T, Dean PB, Tabar L, Tot T, Dean PB (2005). Introduction - The normal breast: comparative subgross anatomy and mammography. Breast cancer. The art and science of early detection with mammography.

[2] McCormack VA, dos Santos Silva I (2006). Breast density and parenchymal patterns as markers of breast cancer risk: a meta-analysis. Cancer Epidemiol Biomarkers Prev..

[3] (2008). Female Breast Cancer Incidence in Peninsula Malaysia 2003–2005. The 3rd Report of the National Cancer Registry. Ministry of Health, Malaysia.

[4] Olsen AH, Bihrmann K, Jensen MB, Vejborg I, Lynge E (2009). Breast density and outcome of mammographic screening: a cohort study. Br J Cancer.

[5] Maskarinec G, Pagano I, Lurie G, Wilkens LR, Kolonel LN (2005). Mammographic density and breast cancer risk: the multricentric cohort study. Am J Epidemiol.

[6] Torres-Mejía G, De Stavola B, Allen DS, Pérez-Gavilán JJ, Ferreira JM, Fentiman IS, Dos Santos Silva I (2005). Mammographic features and subsequent risk of breast cancer: a comparison of qualitative and quantitative evaluations in the guernsey prospective studies. Cancer Epidemiol Biomarkers Prev.

[7] Vavek PM, Geller BM (2004). A prospective study of breasrt cancer risk using routine mammographic breast density measurements. Cancer Epidemiol Biomarkers Prev.

[8] Ziv E, Tice J, Smith-Bindman R, Shepherd J, Cummings S, Kerlikowske K (2004). Mammographic density and estrogen receptor status of breast cancer. Cancer Epidemiol Biomarkers Prev.

[9] Tabar L, Tot T, Dean PB, Tabar L, Tot T, Dean PB (2005). Pattern IV. Breast cancer. The art and science of early detection with mammography.

[10] Jakes RW, Duffy SW, Ng FC, Gao F, Ng EH (2000). Mammographic parenchymal patterns and risk of breast cancer at and after a prevalence screen in Singaporean women. Int J Epidemiol.

[11] Gram IT, Funkhouser E, Tabár L (1997). The Tabár classification of mammographic parenchymal patterns. Eur J Radiol.

[12] Cancer Registry of Norway [online].

[13] Kopans DB, Kopans DB (2007). Breast anatomy and basic histology, physiology, and pathology. Breast Imaging.

[14] Gram IT, Bremnes Y, Ursin G, Maskarinec G, Bjurstam N, Lund E (2005). Percentage density, Wolfe’s and Tabar’s Mammographic Patterns: Agreement and Association with Risk Factors for Breast Cancer. Breast Cancer Research.

[15] Faizatuddarain M, Toh ST, Nik Nasri I, Kamarulzaman I, Wan Rosmanira I, Zulfiqar A (2003). Effects of Hormone Replacement on the Breast. Asian Oceanian Journal of Radiology.

